# Supplementary figures and images for: Prediction of overt hepatic encephalopathy by the continuous reaction time method and the portosystemic encephalopathy syndrome test in clinically mentally unimpaired patients with cirrhosis
Source: PLoS One. 2019 Dec 12;14(12):e0226283. doi: 10.1371/journal.pone.0226283 (PMC6907801; doi:10.1371/journal.pone.0226283)

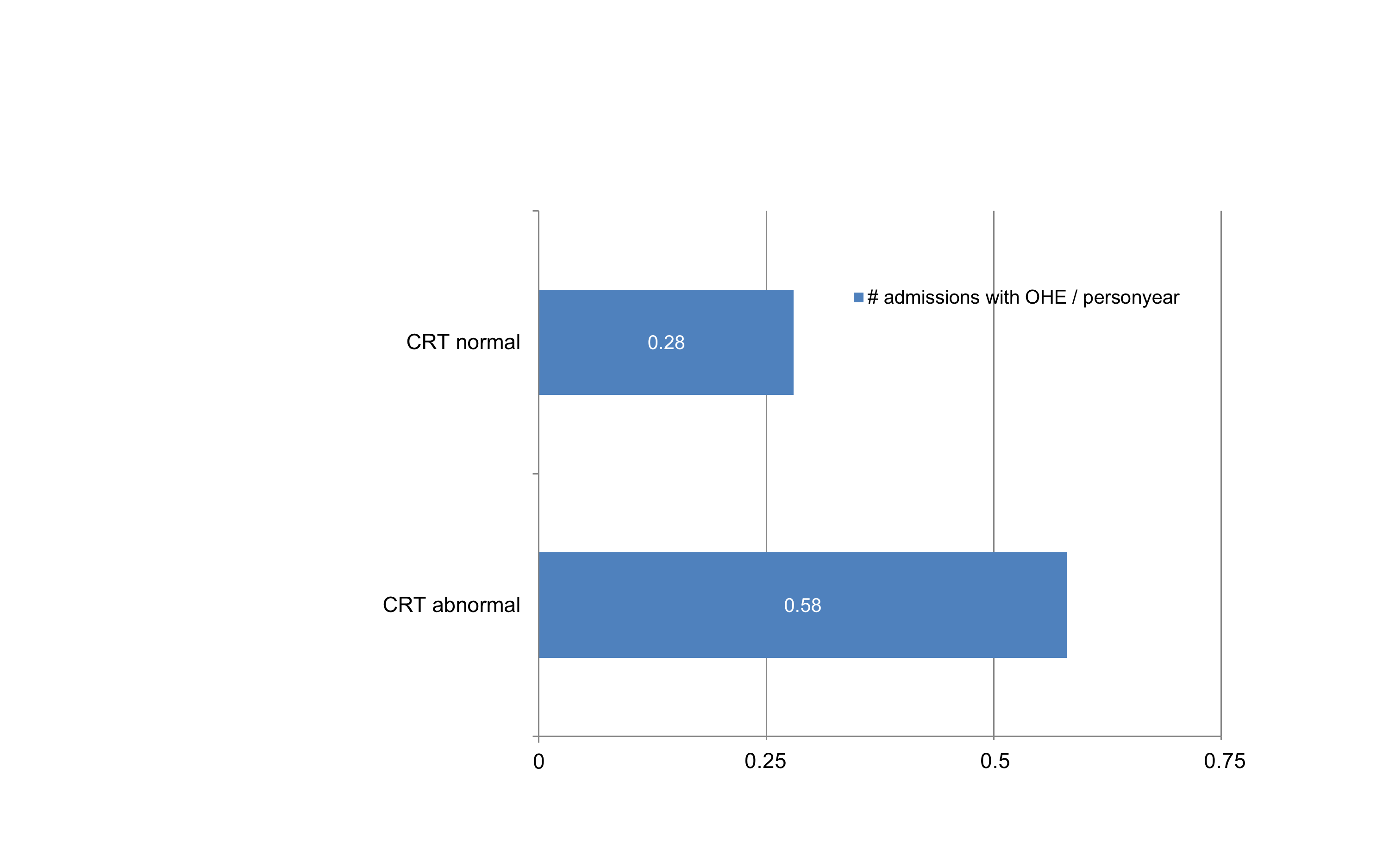

Supplement: S1 Fig — Disregarding psychometric hepatic encephalopathy score. (TIF) [file pone.0226283.s002.tif]

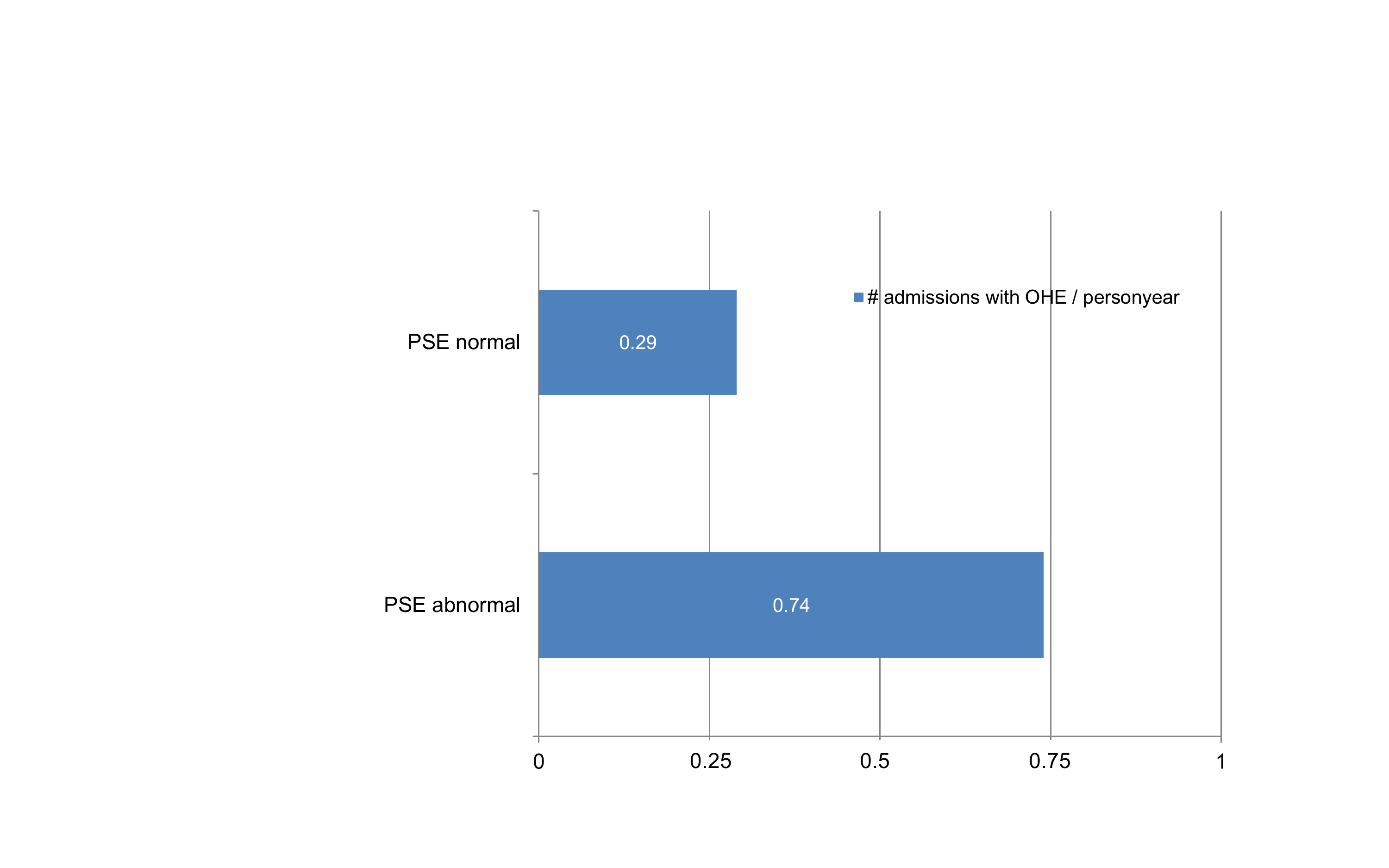

Supplement: S2 Fig — Disregarding CRT index. (TIF) [file pone.0226283.s003.tif]
