# Supplementary material for: Prediction of overt hepatic encephalopathy by the continuous reaction time method and the portosystemic encephalopathy syndrome test in clinically mentally unimpaired patients with cirrhosis
Source: PLoS One. 2019 Dec 12;14(12):e0226283. doi: 10.1371/journal.pone.0226283 (PMC6907801; doi:10.1371/journal.pone.0226283)
Supplement: S1 Table — (DOCX) [file pone.0226283.s004.docx]

**S1 Table. Psychometric test results in patients with: no OHE event, a single OHE event and recurring OHE events**

|  | **No OHE episodes** | **One OHE episode** | **>1 OHE episode** | **P-value ANOVA** |
| --- | --- | --- | --- | --- |
| CRT index, mean (SE; 95% Cl) | 1.9 (0.08;1.8-2.1) | 1.8 (0.12;1.6-2.1) | 1.7 (0.14; 1.4-2.0) | 0.45 |
| PHES, mean (SE; 95% Cl) | -3.6 (0.47; -4.6 - -2.7) | -4.5 (0.94; -6.4 - -2.7) | -5.2 (1.2; -7.6 - -2.7) | 0.69 |

A cohort of 130 patients with liver cirrhosis, tested for the presence of discrete cognitive deficits using CRT and PSE test.

Data is expressed as mean, SE= standard error and 95% confidence interval.

Abbreviations: OHE: Overt hepatic encephalopathy, CRT index : (score given after CRT): abnormal if below 1.9, PHES psychometric hepatic encephalopathy score (score given after PSE): abnormal if below -4
