# Supplementary material for: Prediction of overt hepatic encephalopathy by the continuous reaction time method and the portosystemic encephalopathy syndrome test in clinically mentally unimpaired patients with cirrhosis
Source: PLoS One. 2019 Dec 12;14(12):e0226283. doi: 10.1371/journal.pone.0226283 (PMC6907801; doi:10.1371/journal.pone.0226283)
Supplement: S2 Table — Supplementary statistical output—for linear logistic regression model with margins. Supplementary information to Fig 2. (DOCX) [file pone.0226283.s005.docx]

**S2 Table. Analysis of association: Multivariable linear regression analysis on OHE admission per person year, coefficients and confidence intervals**

|  |  |  |  |  |  |
| --- | --- | --- | --- | --- | --- |
| **OHE per year in study** | **Coef.** | **Std. Err.** | **t** | **P>t** | **95% Conf. interval** |
| *Testgroup* |  |  |  |  |  |
| CRT abnormal and PSE normal | .067 | .15 | 0.44 | 0.66 | -.23 - .37 |
| CRT normal and PSE abnormal | .132 | .35 | 0.38 | 0.71 | -.56 - .82 |
| CRT and PSE abnormal | .301 | .28 | 1.08 | 0.28 | -.25 - .86 |
| *Adjustment for potential confounder* |  |  |  |  |  |
| Age | .017 | .01 | 1.66 | 0.10 | -.003- . 04 |
| MELDscore | .003 | .01 | 0.33 | 0.74 | -.017- .02 |
| Gender | -.160 | .22 | -0.72 | 0.47 | -.59 - .28 |
| Child Pugh | .198 | .07 | 2.74 | 0.01 | .055 - .34 |
| _cons | -2.015 | .92 | -2.19 | 0.031 | -3.83 - -.19 |

| **Testgroup** | **Margin** | **Std. Err.** | **t** | **P>t** | **95% Conf. Interval** |
| --- | --- | --- | --- | --- | --- |
| CRT and PSE normal | .205 | .076 | 2.6 | 0.009 | .053 - .35 |
| CRT abnormal and PSE normal | .389 | .13 | 2.9 | 0.004 | .12 - .64 |
| CRT normal and PSE abnormal | .539 | .35 | 1.5 | 0.131 | -.16 - 1.24 |
| CRT and PSE abnormal | .812 | .32 | 2.4 | 0.015 | .16 - 1.46 |

Supplementary statistical output for linear logistic regression model with margins. Supplementary information to Fig 2 The number of admissions with overt hepatic encephalopathy (OHE), per person year, in the four test groups.
